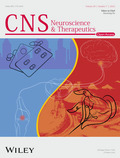# Front cover

**DOI:** 10.1111/cns.14369

**Published:** 2023-07-11

## Abstract

The cover image is based on the Original Article *Modulation effects of different treatments on periaqueductal gray resting state functional connectivity in knee osteoarthritis knee pain patients* by Jun Zhou et al., https://doi.org/10.1111/cns.14153.